# Mcl‐1 inhibition overcomes BET inhibitor resistance induced by low FBW7 expression in breast cancer

**DOI:** 10.1111/jcmm.17210

**Published:** 2022-02-07

**Authors:** Xu Wang, Xiaolin Wei, Yu Cao, Peng Xing

**Affiliations:** ^1^ Department of Breast Surgery The First Affiliated Hospital of China Medical University Shenyang China

**Keywords:** BET inhibitor resistance, breast cancer, FBW7, Mcl‐1

## Abstract

While the promise of bromodomains and extraterminal (BET) protein inhibitors (BETis) is emerging in breast cancer (BC) therapy, resistance in these cells to BETis conspicuously curbs their therapeutic potential. FBW7 is an important tumour suppressor. However, the role of FBW7 in BC is not clear. In the current study, our data indicated that the low expression of FBW7 contributes to the drug resistance of BC cells upon JQ1 treatment. shRNA‐mediated FBW7 silencing in FBW7 WT BC cells suppressed JQ1‐induced apoptosis. Mechanistically, it was revealed that this diminished FBW7 level leads to Mcl‐1 stabilization, while Mcl‐1 upregulation abrogates the killing effect of JQ1. Mcl‐1 knockdown or inhibition resensitized the BC cells to JQ1‐induced apoptosis. Moreover, FBW7 knockdown in MCF7 xenografted tumours demonstrated resistance to JQ1 treatment. The combination of JQ1 with a Mcl‐1 inhibitor (S63845) resensitized the FBW7 knockdown tumours to JQ1 treatment *in vivo*. Our study paves the way for a novel therapeutic potential of BETis with Mcl‐1 inhibitors for BC patients with a low FBW7 expression.

## 1 INTRODUCTION

Globally, breast cancer (BC) has emerged as a ubiquitous malignancy and predominant cause of death in women.^1^ While their survival has been augmented by early detection and adjuvant therapy, advanced BC remains challenging due to the limited efficacy of current therapies.^2^ The clinically vital categorization of BC ubiquitously is based on the expression of the estrogen receptor (ER) and the progesterone receptor (PR) and the human epidermal growth factor receptor 2 (HER2) amplification status.^3^ This categorization guides the first‐line of the therapeutic approach that is adopted.^4^, ^5^ However, a challenge emerges for triple‐negative breast cancer (TNBC) patients, where all three of these receptors are absent with no FDA‐approved targeted therapies yet available, necessitating traditional cytotoxic chemotherapy as the only solution.^6^

Epigenetic regulators have emerged as being resourceful for treating hematologic malignancies and solid tumours.^7^ The crucial involvement of the bromodomain and extraterminal (BET) family of proteins in gene expression via recruitment of transcriptional proteins is known.^8^ Targeting these proteins by employing BET inhibitors (BETis) has recently emerged as a potent approach to quell malignancies^9^ with documentation of its efficacy in various tumours, including breast cancer.^10^ Apoptosis is one of the major mechanisms for the killing effect exerted by BETis in cancer cells.^11^, ^12^ The involvement of the GSK3β/Fbw7/proteasome pathway has also been reported in BETi‐mediated cell death in various cancers.^13^, ^14^ However, the involvement of this pathway in the therapeutic effect of BETis in BC remains to be corroborated.

The tumour‐suppressive functioning of FBW7 in several mouse and human cancers is known.^15^, ^16^ It is an F‐box protein comprising a key element of the SCF (SKP1‐CUL1‐F‐box) E3 ligase complex that functions in protein ubiquitination and degradation,^17^ including cyclin E1, c‐Jun, c‐Myc, Notch1 and Mcl‐1.^18^ The inactivation of FBW7 in numerous human malignancies entails gene mutation^19^, ^20^ and downregulation.^21^ While BC rarely demonstrates *FBW7* mutations,^22^ the *FBW7* genetic locus is frequently deleted in TNBC,^23^ and the FBW7 promoter is highly hypermethylated in 51% of primary BC tumours.^24^ Loss of FBW7 expression has been documented in several breast carcinomas and is associated with poor a prognosis.^25^ This is suggestive of the vital role of FBW7 in BC. Furthermore, the involvement of FBW7 in several distinct signaling pathways facilitates its use as a plausible and attractive therapeutic target for BC.

This work entailed probing FBW7 functioning with respect to its impact on BETi therapy in BC samples. Our results suggest the involvement of diminished FBW7 expression in the BETi resistance observed in BC cell lines. The lowered FBW7 expression was shown to lead to Mcl‐1 upregulation at the posttranscriptional level, which accounts for the resistance. Depletion or inhibition of Mcl‐1 was observed to resensitize the BC cells to BETi‐induced apoptosis, which is indicative of the use of targeting Mcl‐1 to plausibly address the BETi resistance caused by the lowered FBW7 expression in BC.

## 2 METHODS AND MATERIALS

### 2.1 Patient samples

Breast cancer samples (*n* = 31) and adjacent normal tissues (*n* = 20) utilized in this study were obtained from the First Affiliated Hospital of China Medical University with complete clinicopathological data. All studies were approved by the Ethics Committee of China Medical University, and informed consent was obtained from all patients.

### 2.2 Cell lines and reagents

The breast cancer cell lines MCF‐7, MDA‐MB‐231, MDA‐MB‐468 and HCC1954 were maintained in RPMI‐1640 plus GlutaMAX‐1 (Gibco) supplemented with 10% fetal calf serum (FCS) and 10 µg/ml insulin. HEK293T cells were maintained in DMEM (Gibco) plus 10% FCS. JQ1, I‐BET762 and I‐BET151 were purchased from Cayman Chemical (Ann Arbor, MI, USA). Trametinib, olaparib, palbociclib, alisertib, dasatinib, 17‐AAG, lapatinib, trastuzumab, cycloheximide (CHX) and Mcl‐1 inhibitors (S63845 and TW‐37) were commercially sourced from Selleckchem (Houston, TX, USA).

### 2.3 Cell viability and apoptosis

For viability assays, cells were plated in 96‐well plates at 1 × 10^4^ cells/ml in RPMI‐1640 medium (Gibco) supplemented with 10% FCS and 10 µg/ml insulin followed by JQ1 treatment. Cell viability was determined by MTT assays (Promega, Madison, WI, USA) based on the manufacturer's protocol. For apoptosis analysis, Hochst‐33258‐based staining was employed to probe apoptosis as described previously.^26^, ^27^

### 2.4 Plasmids and transfections

Short hairpin RNA (shRNA) transfection and subsequent screening were conducted as reported previously.^13^ Lipofectamine 2000 (Thermo Fisher, Waltham, MA, USA) was employed for all transfection assays adhering to the recommendations of the manufacturer. Santa Cruz Biotechnology (Dallas, TX, USA) was the source of the siRNA for Mcl‐1, while the pTOPO‐Mcl‐1 plasmid was sourced from Addgene (#21605). For FBW7 shRNA transfection, the pLKO.1 FBW7 shRNA was purchased from Horizon Discovery (Waterbeach, UK).

### 2.5 Real‐time PCR

The real‐time PCR assay was performed as described in previous studies.^28^, ^29^ Briefly, total RNA was extracted using TRIzol RNA extraction reagent (Invitrogen) and treated with DNaseI. DNA‐free RNA was reverse transcribed using an RNA‐to‐cDNA kit (Invitrogen). Total cDNA was amplified using iTaq Universal SYBR Green Supermix (Bio‐Rad). The results were normalized to GAPDH expression. The primers used for this study included: GAPDH, F: 5′‐GAAGGTGAAGGTCGGAGTC‐3′ and R: 5′‐GAAGATGGTGATGGGATTTC‐3′; and Mcl‐1, F: 5′‐CCAAGGCATGCTTCGGAAA‐3′ and R: 5′‐TCACAATCCTGCCCCAGTTT‐3′.

### 2.6 Western blotting

The western blotting assay was performed as described in previous studies.^30^, ^31^ Primary antibodies against FBW7 (Santa Cruz Biotechnology), PUMA, Bcl‐XL, Bax, β‐actin, cleaved caspase‐3 and Mcl‐1 (Cell Signaling Technology, Danvers, MA) were used. Secondary antibodies were purchased from Cell Signaling Technology. The primary (1:1000) and secondary (1:4000) antibodies were diluted in 2.5% BSA/Tris‐buffered saline with Tween 20 (TBS‐T).

### 2.7 Immunoprecipitation (IP)

After treatment, the indicated cells were collected and resuspended in RIPA buffer (50 mM Tris‐HCl, pH 7.5, 0.5% Nonidet P‐40, 100 mM NaCl) supplemented with a protease inhibitor cocktail (Invitrogen). The cell suspension was sonicated and centrifuged at 13,000 g for 20 min to prepare cell lysates. For the IP experiment, the IP antibody (1 mg) was incubated with protein G/A‐agarose beads (Invitrogen) for 20 min at room temperature. The beads were washed twice with PBS containing 0.02% Tween‐20, incubated with cell lysates on a rocker for 4 h at room temperature, and then washed with PBS three times. Beads were then boiled in 2× Laemmli sample buffer and subjected to SDS‐PAGE and western blotting.

### 2.8 Xenograft mouse model

The housing of the animals (5‐ to 6‐week‐old female Nu/Nu mice) was in a sterile environment in micro isolator cages with access to water and chow *ad libitum*. MCF7 cells (4 × 10^6^) stably transfected with control or FBW7 shRNA were subcutaneously injected on the right. After tumour growth for 7 days, the mice were treated with JQ1 (i.p.; 30 mg/kg every other day for 10 days). For the combination treatment. MCF7 cells (4 × 10^6^) stably transfected with FBW7 shRNA were subcutaneously injected on the right. After tumour growth for 7 days, mice were treated with JQ1 (i.p.; 30 mg/kg every other days for 10 days) and/or Mcl‐1 inhibitor (S63845, 15 mg/kg intravenously, every 3 day for 10 days). Calipers were employed to measure the tumour size every 2 days, with the formula: 1/2 × length × width^2^ employed for computing the tumour volumes. All animal experiments and research plans were approved by the Animal Research Committee of China Medical University.

### 2.9 Patient‐derived xenograft (PDX) mouse model

The PDX model was established using primary tumours resected from patients with written informed consent and an approved Institutional Review Board agreement. Then, tumours were implanted subcutaneously and passaged. In short, within 4 h after tumour removal, the breast cancer sample from an unidentified patient was transported to the laboratory in Antibiotic/Antimycotic Solution (Invitrogen). Tissues were cut into 25‐mg pieces and directly implanted subcutaneously on both flanks of the NOD. Cg‐*Prkdc^scid^* *Il2rg^tm1Wjl^*/SzJ (NSG) mice. Tumours that were passaged and expanded for two generations (P2) in NSG mice were used for the experiments. Mice were treated with JQ1 (i.p.; 10 mg/kg every other day for 10 days) and/or Mcl‐1 inhibitor (S63845, 15 mg/kg intravenously, every 3 days for 10 days). The caliper was used to monitor the tumour volume, and the tumour volume was calculated as: 1/2 × length × width^2^. The ethical endpoint was defined as the point in time when the tumour reached 2 cm or more in any dimension. The tumour was dissected and fixed in 10% formalin and embedded in paraffin. By using an AlexaFluor 488‐conjugated secondary antibody (Invitrogen) for detection and 4’ 6‐Diamidino‐2‐phenylindole (DAPI) for nuclear counter staining, the 5‐μm paraffin‐embedded tumour sections were subjected to active caspase 3 staining.

### 2.10 Statistical analysis

All assays illustrated in the figures were conducted in duplicate with two or three independent runs. The final figures are inclusive of the representative results. The computation of *p* values entailed was performed by the two‐tailed distribution Student's *t*‐test on paired or unpaired datasets. *p* < 0.05 was considered statistically significant.

## 3 RESULTS

### 3.1 Low FBW7 expression in breast cancer patients

**FIGURE 1:**
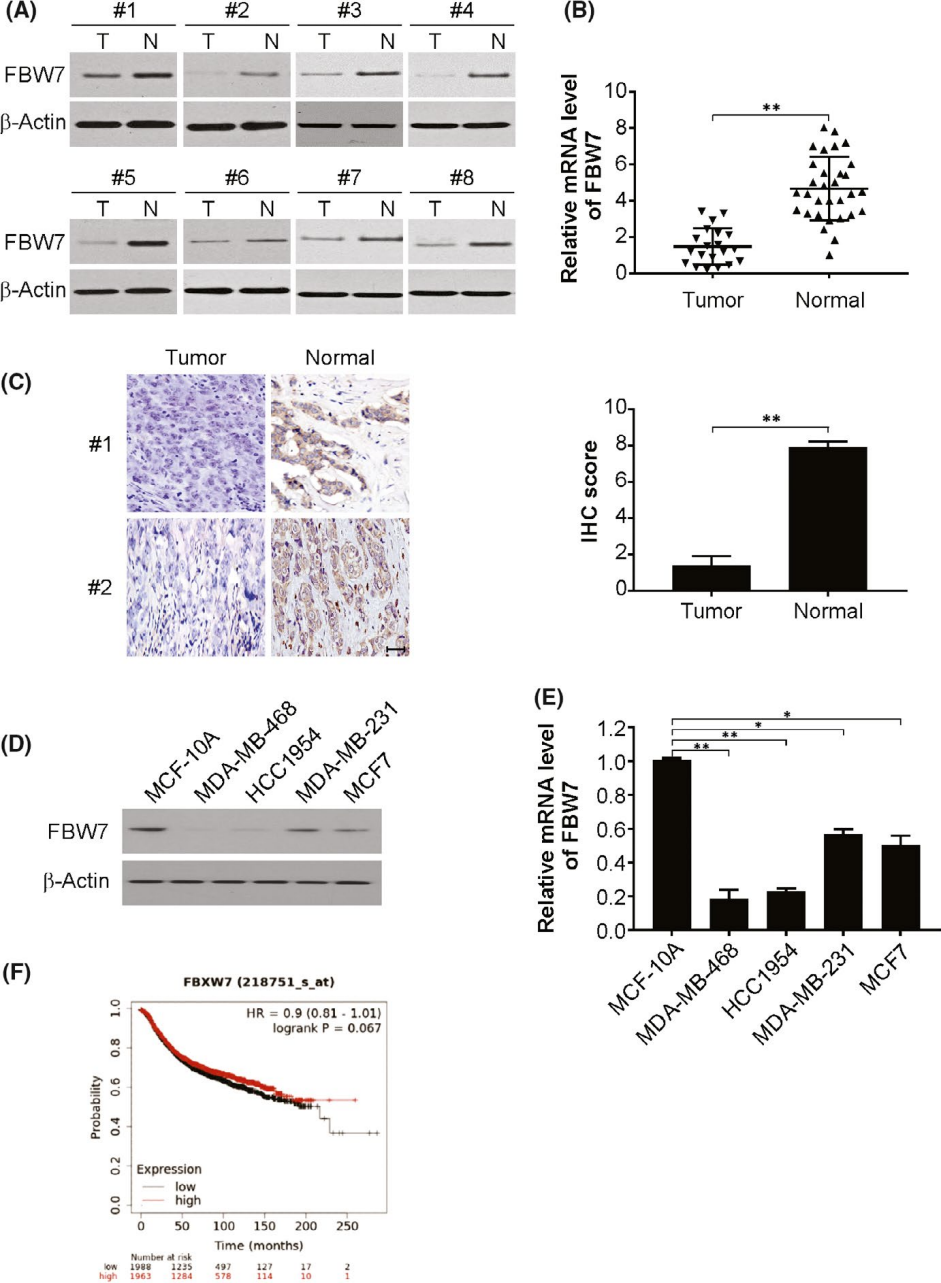
FBW7 is one of the prognostic biomarkers for breast cancer. (A) The protein expression level of FBW7 was analysed by western blotting in breast cancer patient specimens and non‐tumour tissues. (B) The mRNA level of FBW7 was analysed by real‐time PCR in breast cancer patient specimens and non‐tumour tissues. (C) The protein expression level of FBW7 was analysed by IHC in breast cancer patient specimens and non‐tumour tissues. Scale bar: 25 μm. (D) The expression of FBW7 was analysed by western blotting in MCF‐10A and breast cancer cells. (E) The expression of FBW7 was analysed by real‐time PCR in MCF‐10A and breast cancer cells. (F) The correlation of FBW7 expression with BC patient survival was analysed by Kaplan‐Meier plotter. Data presented as Means ± SD (*n* = 3). **p < 0.01

To determine the functional role of FBW7 in BC, we first probed the expression of FBW7 in clinical breast cancer specimens, including 31 breast cancer tissues and 20 adjacent nontumour tissues. Western blotting and real‐time PCR analysis demonstrated that FBW7 protein expression was decreased in malignant tissues compared to adjacent nontumour tissues (Figure 1A and B). Furthermore, this lowered FBW7 level in the malignant samples was also demonstrated by IHC analysis (Figure 1C). Our findings were also indicative of diminished FBW7 expression in BC cells vs. MCF‐10A cells, which was detected by western blotting and real‐time PCR (Figure 1D and E). Moreover, higher expression of FBW7 displayed a correlation with better survival in BC patients, as evidenced by the database analysis from the km plot (http://kmplot.com/) (Figure 1F). These results demonstrate the plausible involvement of FBW7 in BC progression.

### 3.2 FBW7 decreases breast cancer cell growth in vitro and in vivo

**FIGURE 2:**
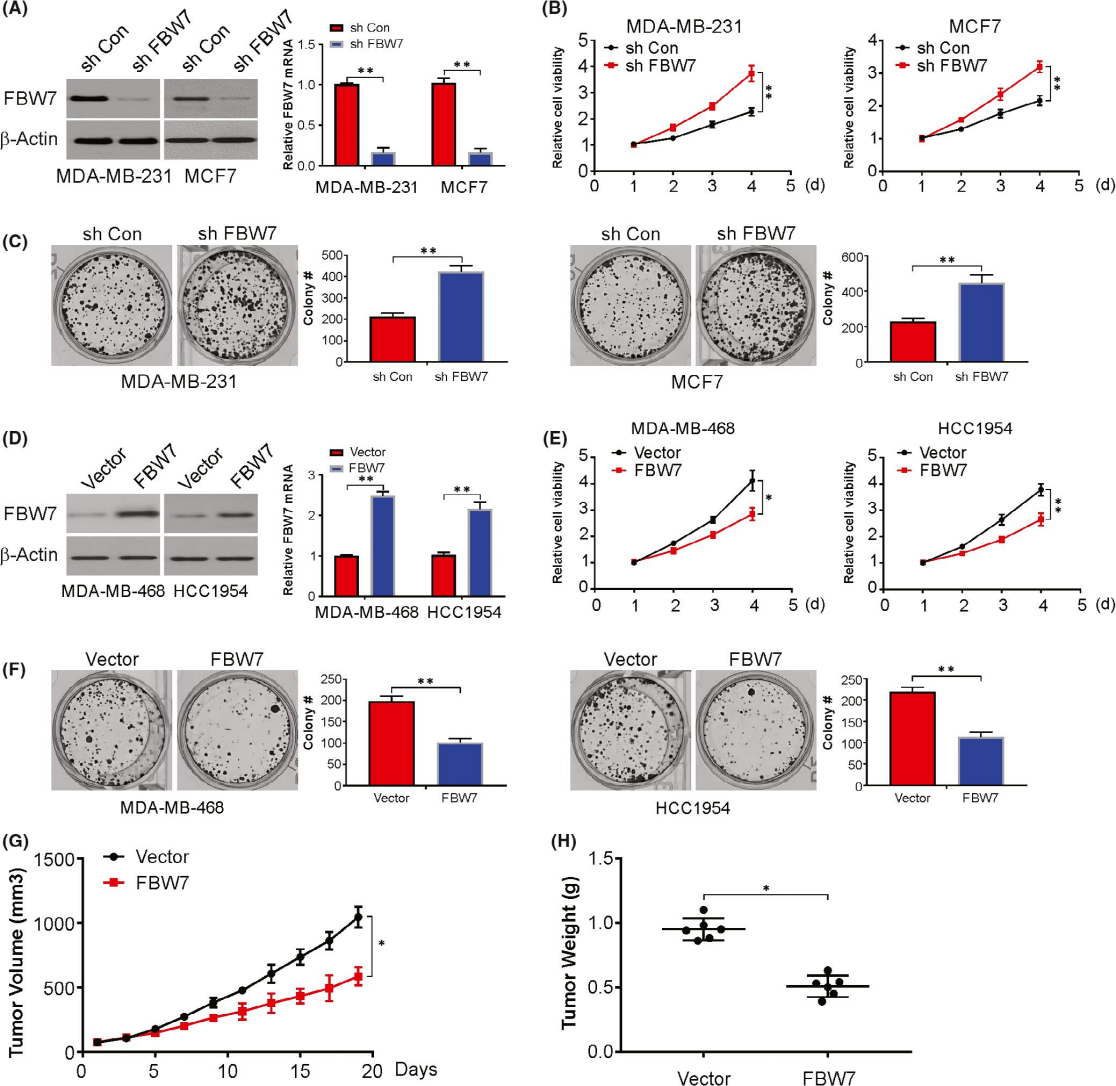
FBW7 suppresses breast cancer growth in vitro and in vivo. (A) MDA‐MB‐231 and MCF‐7 cells were transfected with sh RNA against FBW7, FBW7 expression was analysed by western blotting (*Left*) and real‐time PCR (*Right*). (B) The viability of MDA‐MB‐231 (*Left*) and MCF‐7 (*Right*) cells with sh FBW7 transfection was analysed by MTT. (C) Colony formation assay of MDA‐MB‐231 (*Left*) and MCF‐7 (*Right*) cells with sh FBW7 transfection. (D) MDA‐MB‐468 and HCC1954 cells were transfected with FBW7 overexpression plasmid, FBW7 expression was analysed by western blotting (*Left*) and real‐time PCR (*Right*). (E) The viability of MDA‐MB‐468 (*Left*) and HCC1954 (*Right*) cells with FBW7 overexpression plasmid transfection was analysed by MTT. (F) Colony formation assay of MDA‐MB‐468 (*Left*) and HCC1954 (*Right*) cells with FBW7 overexpression plasmid transfection. (G) and (H) MDA‐MB‐468 cells with or without FBW7 construct transfected were injected subcutaneously into the nude mice for xenografts assay for 19 days. The tumour volume (G) and weight (H) of xenograft was determined. Data presented as Means ± SD (*n* = 3). *p < 0.05; **p < 0.01

We next considered whether FBW7 had any effect on the biological behavior of breast cancer cells. As shown in Figure 1D, FBW7 was highly expressed in MCF7 and MDA‐MB‐231 cells. In contrast, the expression level of FBW7 was lower in MDA‐MB‐468 and HCC1954 cells. We suppressed the expression levels of FBW7 in MCF7 and MDA‐MB‐231 cells using specific short hairpin RNA (shRNA) (Figure 2A). Cell growth ability was determined by MTT assay and colony formation assay after knocking down FBW7 in these two cancer cell lines (Figure 2B and C). In contrast, overexpression of FBW7 in BC inhibited cell growth (Figure 2D–2F). Our findings demonstrate that FBW7 knockdown markedly promotes breast cancer proliferation in vitro. Next, the in vivo growth was scored in the xenograft tumour assay. A marked decrease in growth was documented by FBW7 overexpression (Figure 2G and H). The above data indicate that FBW7 overexpression suppresses tumour growth in BC.

### 3.3 High expression of FBW7 contributes to BET inhibitor sensitivity in breast cancer cell lines

**FIGURE 3:**
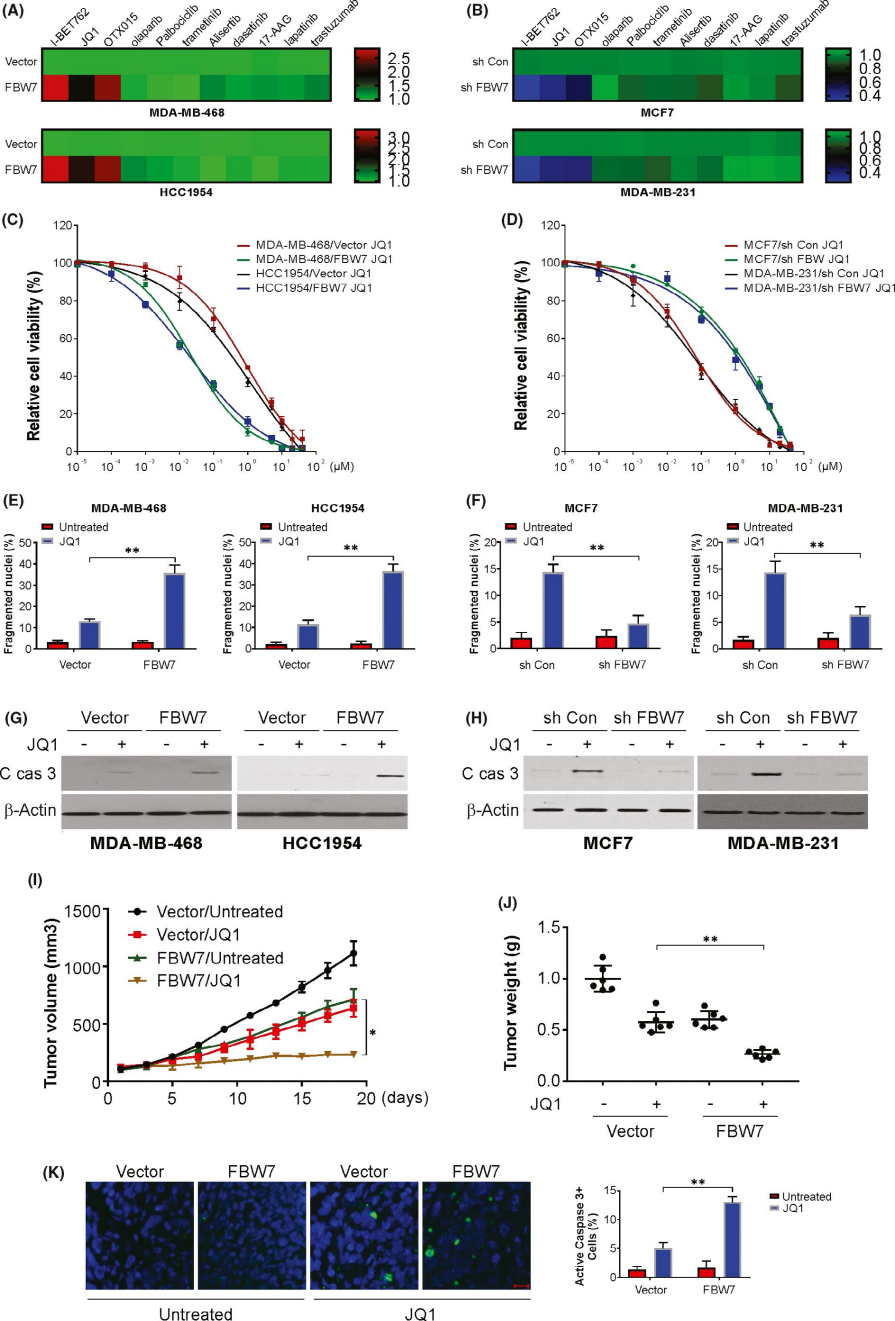
FBW7 regulates the sensitivity of BETis in breast cancer cells. (A) MDA‐MB‐468 (*Upper*) and HCC1954 (*Lower*) cells with FBW7 overexpression plasmid transfection were treated with indicated drugs for 72 h. The IC50 values was analysed and IC50 ratio of FBW7 vs. vector were determined and shown in heatmap. (B) MDA‐MB‐231 (*Upper*) and MCF‐7 (*Lower*) cells with FBW7 sh RNA transfection were treated with indicated drugs for 72 h. The IC50 values was analysed and IC50 ratio of FBW7 vs. vector were determined and shown in heatmap. (C) MDA‐MB‐468 and HCC1954 cells with FBW7 overexpression plasmid transfection were treated with increasing dose of JQ1, cell viability was determined by MTT. (D) MDA‐MB‐231 and MCF‐7 cells with FBW7 sh RNA transfection were treated with increasing dose of JQ1, cell viability was determined by MTT. (E) MDA‐MB‐468 (*Left*) and HCC1954 (*Right*) cells with FBW7 overexpression plasmid transfection were treated with 5 µM JQ1 for 24 h. The apoptosis was analysed by Hochst‐33258 staining. (F) MDA‐MB‐231 (*Left*) and MCF‐7 (*Right*) cells with FBW7sh RNA transfection were treated with 5 µM JQ1 for 24 h. The apoptosis was analysed by Hochst‐33258 staining. (G) MDA‐MB‐468 and HCC1954 cells with FBW7 overexpression plasmid transfection were treated with 5 µM JQ1 for 24 h. Active caspase 3 was determined by western blotting. (H) MDA‐MB‐231 and MCF‐7 cells with FBW7sh RNA transfection were treated with 5 µM JQ1 for 24 h. Active caspase 3 was determined by western blotting. (I) MDA‐MB‐468 cells with or without FBW7 construct transfected were injected subcutaneously into the nude mice. These mice were treated with or without JQ1 for 10 days. Tumour volume was determined. (J) Tumour weight was determined. (K) Mice with xenograft tumours were treated with JQ1 or the vehicle as in (I) for 4 consecutive days. Paraffin‐embedded sections of tumour tissues were analysed by cleaved caspase 3 staining. Scale bar: 25 μm. Data presented as Means ± SD (*n* = 3). *p < 0.05; **p < 0.01

To further scrutinize the impacts of FBW7 in BC, we probed the sensitivity of cell lines to small molecule drugs with FBW7 knockdown or overexpression (Figure 3A and B). This entailed an initial treatment of FBW7 knockdown or overexpressing BC cells with several small molecules, followed by analysis of the drug sensitivity by computing the IC50 for each group. The normalized IC50 values of these inhibitors in the FBW7 knockdown/overexpressed group vs. those in the control group are illustrated as a heatmap (Figure 3A and B). MDA‐MB‐468 and HCC1954 cells demonstrated sensitivity to BETis after FBW7 overexpression (Figure 3A and B). The opposite trend was observed for the cells with the FBW7 knockdown, with resistance to BETis observed in MCF7 and MDA‐MB‐231 cells (Figure 3A and B). The corroboration of FBW7 functioning in the sensitivity of BC cells to BETis entailed the treatment of cells overexpressing FBW7 with JQ1. MTT assays demonstrated the augmented suppression of BC cell growth by JQ1 in the FBW7 overexpression group vs. the control group (Figure 3C). FBW7 knockdown in BC cells demonstrated resistance to JQ1 compared to that in BC cells transfected with the sh control (Figure 3D).

Furthermore, the overexpression of FBW7 caused augmented apoptosis in malignant cells after JQ1 treatment, which was determined by fragmented nuclei and caspase 3 activation (Figure 3E and G). The absence of FBW7 in MCF7 cells also suppressed apoptosis induced by JQ1 (Figure 3F and H). This hampered malignant cell growth was also documented in the *in vivo* assays (Figure 3J–K), with increased apoptosis in the FBW7 overexpression group treated with JQ1 compared to the control group treated with only JQ1. Therefore, our data suggest the impact of FBW7 expression on the sensitivity of BC cells to BETis.

### 3.4 FBW7 mediates the degradation of Mcl‐1 in BC cells

**FIGURE 4:**
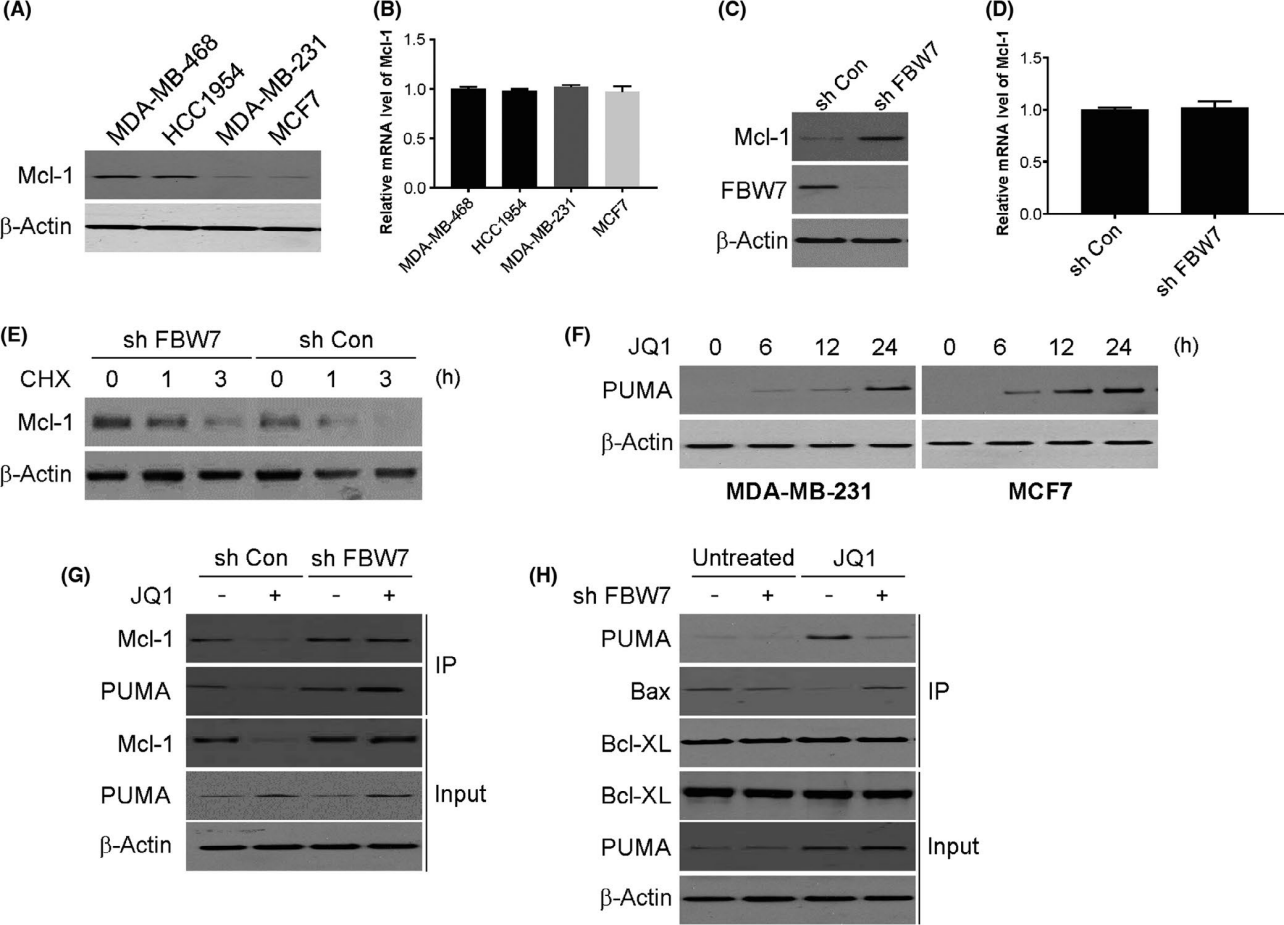
FBW7‐mediated Mcl‐1 degradation is required for the sensitivity of JQ1. (A) Mcl‐1 expression in indicated cell lines was determined by western blotting. (B) Mcl‐1 mRNA level in indicated cell lines was determined by real‐time PCR. (C) Mcl‐1 expression in FBW7 knockdown cells was determined by western blotting. (D) Mcl‐1 mRNA level in FBW7 knockdown cells was determined by real‐time PCR. (E) The MCF‐7 transfected with FBW7 shRNA were treated with cycloheximide as indicated time. The expression of Mcl‐1 was analysed by western blotting. (F) MDA‐MB‐231 and MCF‐7 cells were treated with JQ1 at indicated time points. PUMA expression was determined by western blotting. (G) MDA‐MB‐231 cells with or without FBW7 sh RNA transfection were treated with JQ1 for 24 h. IP was performed to pull down Mcl‐1, followed by western blotting of indicated proteins. (H) MDA‐MB‐231 cells with or without FBW7 sh RNA transfection were treated with JQ1 for 24 h. IP was performed to pull down Bcl‐XL, followed by western blotting of indicated proteins

Given reports of degradation of the anti‐apoptotic protein Mcl‐1 by FBW7,^32^ the impact of the latter on Mcl‐1 expression in BC cells was probed next. An initial screening of Mcl‐1 levels in the aforementioned BC cell lines revealed a negative correlation of Mcl‐1 protein with FBW7 expression in all four cell lines (Figure 4A). However, no such variation was documented for the Mcl‐1 mRNA level in these BC cells (Figure 4B). While FBW7 knockdown in MCF7 cells augmented Mcl‐1 protein levels (Figure 4C), the Mcl‐1 mRNA did not demonstrate any such change (Figure 4D). Furthermore, the depletion of FBW7 extended the half‐life of Mcl‐1 in MCF7 cells upon cycloheximide (CHX) treatment (Figure 4E). Therefore, our data suggest that Mcl‐1 is a plausible downstream target of FBW7 in mediating apoptosis induced by BETis in BC cells. As earlier work demonstrated PUMA induction by BETis in CRC cells,^33^ we then assessed the effect of JQ1 on PUMA in BC. The induction of PUMA by JQ1 in MCF7 cells was observed (Figure 4F). In *FBW7*‐KD cells, following JQ1 treatment, Mcl‐1 binding to PUMA was augmented, causing a lowered interaction of the latter with Bcl‐XL, while the Bax‐Bcl‐XL interaction was increased (Figure 4G and H). These observations therefore demonstrate Mcl‐1 upregulation is due to the loss of FBW7, which leads to PUMA induction via JQ1 to cause resistance to JQ1 in BC.

### 3.5 Inhibition or depletion of Mcl‐1 sensitizes BC cells to BET inhibitors

**FIGURE 5:**
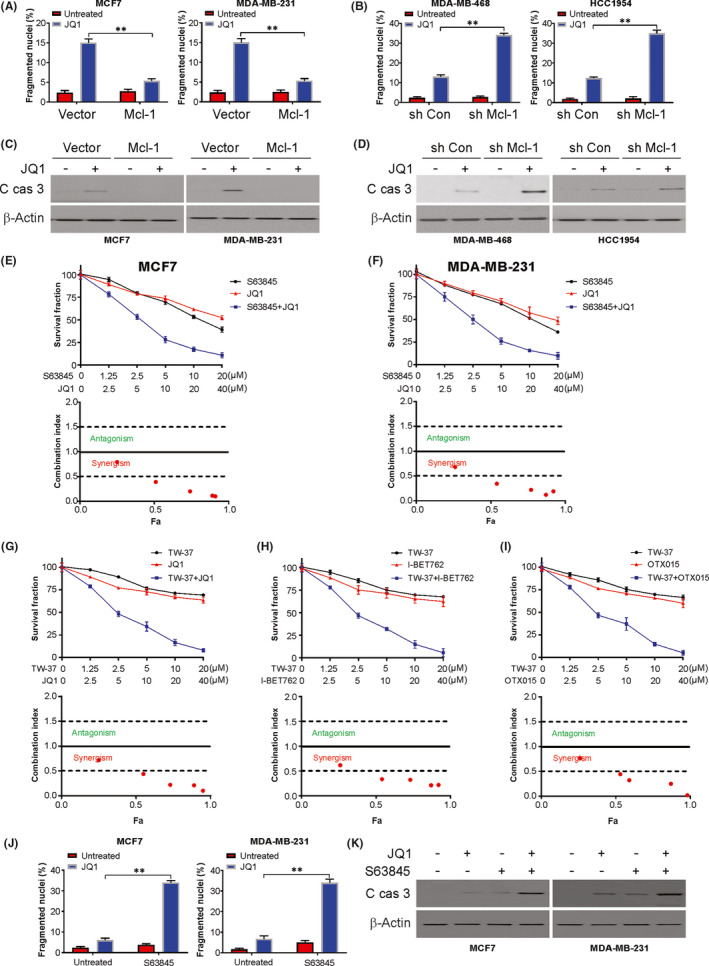
Mcl‐1 depletion or inhibition sensitized the BC cells to BETis induced apoptosis. (A) MCF‐7 (*Left*) and MDA‐MB‐231 (*Right*) cells with or without Mcl‐1 overexpression were treated with 5 µM JQ1 for 24 h. The apoptosis was analysed by Hochst‐33258 staining. (B) MDA‐MB‐468 (*Left*) and HCC1954 (*Right*) cells with or without Mcl‐1 sh RNA transfection were treated with 5 µM JQ1 for 24 h. The apoptosis was analysed by Hochst‐33258 staining. (C) MCF‐7 (*Left*) and MDA‐MB‐231 (*Right*) cells with or without Mcl‐1 overexpression were treated with 5 µM JQ1 for 24 h. Active caspase 3 was determined by western blotting. (D) MDA‐MB‐468 (*Left*) and HCC1954 (*Right*) cells with or without Mcl‐1 sh RNA transfection were treated with 5 µM JQ1 for 24 h. Active caspase 3 was determined by western blotting. (E) MCF‐7 cells were treated with JQ1 and S63845 at indicated concentration, cell viability was determined by MTT (*Upper*). Combination index (CI) and fraction affected of JQ1 and S63845 combining at different concentrations in MCF‐7 cells were analysed by the CompuSyn program (ComboSyn) (*Lower*). (F) MDA‐MB‐231 cells were treated with JQ1 and S63845 at indicated concentration, cell viability was determined by MTT (*Upper*). Combination index (CI) and fraction affected of JQ1 and S63845 combining at different concentrations in MCF‐7 cells were analysed by the CompuSyn program (ComboSyn) (*Lower*). (G) MCF‐7 cells were treated with JQ1 and TW‐37 at indicated concentration, cell viability was determined by MTT (*Upper*). Combination index (CI) and fraction affected of JQ1 and S63845 combining at different concentrations in MCF‐7 cells were analysed by the CompuSyn program (ComboSyn) (*Lower*). (H) MCF‐7 cells were treated with I‐BET762 and TW37 at indicated concentration, cell viability was determined by MTT (*Upper*). Combination index (CI) and fraction affected of JQ1 and S63845 combining at different concentrations in MCF‐7 cells were analysed by the CompuSyn program (ComboSyn) (*Lower*). (I) MCF‐7 cells were treated with OTX015 and TW‐37 at indicated concentration, cell viability was determined by MTT (*Upper*). Combination index (CI) and fraction affected of JQ1 and S63845 combining at different concentrations in MCF‐7 cells were analysed by the CompuSyn program (ComboSyn) (*Lower*). (J) MCF‐7 (*Left*) or MDA‐MB‐231 (*Right*) cells were treated with JQ1, S63845 or their combination for 24 h. The apoptosis was analysed by Hochst‐33258 staining. (K) MCF‐7 (*Left*) or MDA‐MB‐231 (*Right*) cells were treated with JQ1, S63845 or their combination for 24 h. Active caspase 3 was determined by western blotting. Data presented as Means ± SD (*n* = 3). **p < 0.01

Further probing of Mcl‐1 functioning in BETis‐induced BC cell apoptosis was performed through its overexpression by transfecting the MCF7 cell line with the Mcl‐1 plasmid. The results of the overexpression of Mcl‐1 in MCF7 cells were consistent with the results of the FBW7 knockdown, which showed that JQ‐1 induced apoptosis (Figure 5A) and caspase‐3 cleavage (Figure 5C) were suppressed. The depletion of Mcl‐1 by siRNA re‐sensitized the MCF7 cells transfected with FBW7 siRNA to JQ‐1‐induced apoptosis (Figure 5B) and reinforced the cleavage of caspase‐3 (Figure 5D).

The synergistic effects of JQ1 with the Mcl‐1 inhibitor S63845 were then probed to corroborate these observations. MCF7 and MDA‐MB‐231 cells demonstrated robust synergistic effects of JQ1 with S63845 in the cell viability assays (Figure 5E and F). These effects were also demonstrated for the already well‐characterized BETis (JQ1, I‐BET762, OTX015) and a Mcl‐1 inhibitor (TW‐37) in MDA‐MB‐231 cells (Figure 5G–I). Moreover, cotreatment with S63845 also augmented the killing effect of JQ‐1in MCF7 and MDA‐MB‐231 cells, as evidenced by apoptosis and caspase‐3 cleavage (Figure 5J and K). Collectively, these results suggest the involvement of Mcl‐1 in the FBW7‐mediated apoptosis induced by BETis in BC cells.

### 3.6 Mcl‐1 inhibitor enhances the killing effect of JQ1 in vivo

**FIGURE 6:**
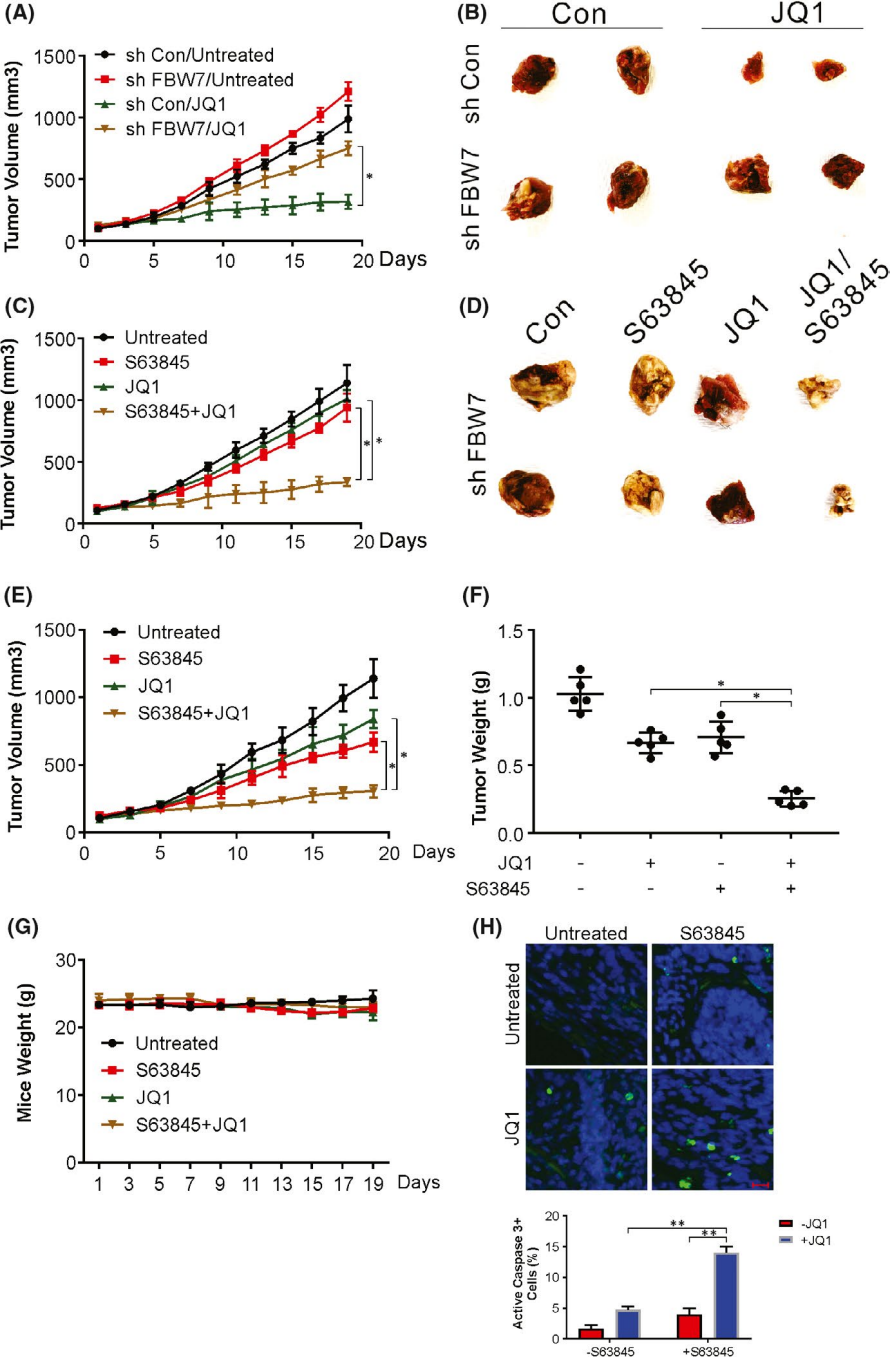
Inhibition of Mcl‐1 enhanced the killing effect of JQ1 in vivo. (A) MCF7 stably transfected with control or FBW7 shRNA were inoculated into nude mice. The tumour growth curves of xenografted tumours with or without JQ1 treatment. (B) The representative tumours. (C) MCF7 stably transfected with FBW7 shRNA were inoculated into nude mice. The tumour growth curves of xenografted tumours with JQ1 in combination with S63845 treatment. (D) The representative tumours. (E) PDX were established in NSG mice. The tumour growth curves of xenografted tumours with JQ1 in combination with S63845 treatment. (F) Tumour weight was determined. (G) Mice weight was determined. (H) Mice with PDX tumours were treated with JQ1 in combination with S63845 as in (E) for four consecutive days. Paraffin‐embedded sections of tumour tissues were analysed by cleaved caspase 3 staining. Scale bar: 25 μm. Data presented as Means ± SD (*n* = 3). *p < 0.05; **p < 0.01

The in vivo assessment of the effect of FBW7 in BETi chemosensitization entailed the use of the athymic nude mouse system harbouring xenograft tumours as outlined in the materials section. Seven days post‐tumour inoculation, the mice were administered JQ1 for 10 days. A conspicuous decrease in MCF7 xenograft tumour growth was observed after JQ1 injection (Figure 6A and B). FBW7 silencing abolished the suppressive effect of JQ1 (Figure 6A and 6B). This was indicative of FBW7 mediating JQ1 sensitivity in vivo. To assess whether Mcl‐1 inhibition could overcome the JQ1 resistance caused by low levels of FBW7, we treated the mice with FBW7 knockdown MCF7 xenografts with JQ1 and an Mcl‐1 inhibitor. Our data showed that this combination with a Mcl‐1 inhibitor re‐sensitized FBW7‐null tumours to JQ1 treatment (Figure 6C and D). In summary, our *in vivo* data demonstrate the critical functioning of FBW7/Mcl‐1 in mediating the chemosensitization and antitumour effects of BETis in BC cells.

The JQ1/S63845 combination was then probed in BC PDX models given the ability of these systems to recapotulate the histology, heterogeneity, and molecular alterations of patient malignancies in a more refined format. The JQ1/S63845 combination, but not JQ1 or S63845 alone, conspicuously diminished PDX tumour growth (Figure 6E and F), with no evident impact on body weight (Figure 6G). The combinatorial system also augmented increased apoptosis in terms of active caspase‐3 levels compared to JQ1 or S63845 alone (Figure 6H). In summary, our in vitro and in vivo data demonstrate the augmented effects of BETis by a Mcl‐1 inhibitor in BC.

## 4 DISCUSSION

This work is suggestive of the involvement of a low level of FBW7 expression in resistance to BETis (here to JQ1 and I‐BET151) in BC cells. Looking at the mechanistic aspects, augmented Mcl‐1 protein levels due to *FBW7* knockdown in BC cells account for the targeting of BETis‐induced apoptosis. The Catalog of Somatic Mutations in Cancer Database documents FBW7 inactivation by a somatic gene mutation in a small subset (~1%) of breast cancers. Nonetheless, *FBW7* gene polymorphisms are linked to high‐stage and ERα‐negative breast cancers.^34^ The results of this work are also consistent with the varied effects of FBW7 expression in TNBC cells. The expression level of FBW7 in BC cells is correlated with the therapeutic effect of BETis in these cells. Overexpression of FBW7 has been shown lead to cell proliferation arrest and apoptosis in BC cells.^35^ Addressing of therapy resistance has also entailed targeting FBW7 in multiple cancers, including BC.^36^, ^37^ Resistance to the BET inhibitors JQ1 and OTX‐015 was documented in T‐ALL tumour cells with FBW7 mutations.^13^ Our work also corroborates of the involvement of FBW7 in BETis sensitivity in BC cells. The lowered expression of FBW7 leads to Mcl‐1 upregulation, which makes the BC cells assessed in our work less sensitive to BETis‐induced apoptosis.

The negative regulation of apoptosis in malignant and healthy cells by Mcl‐1 (of the Bcl‐2 family) is known.^38^ Mcl‐1 has a very short half‐life, and its expression is tightly regulated by its interaction with FBW7, which also mediates its degradation.^32^, ^39^, ^40^ FBW7 mutations in tumours have been shown to increase Mcl‐1 expression to augment resistance to both standard chemotherapy and targeted therapy.^32^, ^38^, ^41^, ^42^ The pathogenesis and poor prognosis arising from elevated Mcl‐1 protein levels in refractory cancers are suggestive of manipulating the protein rather than the mRNA to boost apoptosis and thereby target a malignancy. Given that Mcl‐1 is predominantly involved in resistance to BETis in HCC cells, the use of drugs that down‐regulate Mcl‐1 emerged as promising for augmenting HCC therapy.^43^ The results of this work are also indicative of the inhibition or depletion of Mcl‐1 to overcome BETis resistance in BC cells with a low level of FBW7 expression. In addition, our findings also indicate that JQ1 upregulates PUMA, which promotes apoptosis by releasing Bax from Bcl‐XL, which is consistent with a previous study showing that the BET inhibitor I‐BET151 induces PUMA and sensitizes gliomas to TMZ.^33^ Previous studies have shown that the transcription factor c‐Myc is the target of bromodomain proteins.^44^, ^45^ Furthermore, FBW7 controls proteasome‐mediated degradation of c‐Myc.^46^, ^47^ Thus, c‐Myc may also be involved in FBW7‐mediated BETi resistance in breast cancer. Therefore, targeting Mcl‐1 emerges as a plausible option for BC patients with low levels of FBW7 or with mutated FBW7.

In conclusion, our results suggest the crucial involvement of the FBW7 level in BC cells demonstrating BETi resistance. The inhibition of Mcl‐1 can potentially overcome this roadblock in BETi treatment. This paves the way for the genetic characterization and expression profile of FBW7 to also be taken into account for personalized BC treatment in the future.

## ETHICAL APPROVAL

The study was approved from the Institutional Review Board, the First Affiliated Hospital of China Medical University, Shenyang, China.

## CONFLICT OF INTEREST

The authors declare no conflict of interest.

## AUTHOR CONTRIBUTIONS

**Xu Wang:** Data curation (equal); Formal analysis (equal). **Xiaolin Wei:** Data curation (equal). **Yu Cao:** Data curation (equal). **Peng Xing:** Data curation (equal); Investigation (equal); Project administration (equal).

## Data Availability

The datasets used and/or analysed during the current study are available from the corresponding author on reasonable request.
